# Topics of Public Interest

**Published:** 1913-06

**Authors:** 


### TOPICS OF PUBLIC INTEREST.

*Opening exercises of the Henry Phipps Psychiatric Clinic, Johns Hopkins Hospital, Baltimore, Maryland.*

*Prepared by DR. ARTHUR W. HURD, Buffalo.*

A notable event in the medical world took place April 16-17-18 last, when in Baltimore there was formally opened the psychiatric clinic given to the Johns Hopkins Hospital for the study and treatment of mental diseases. The program was an interesting one, and was participated in by many distinguished physicians, both from this country and abroad. The chief exercises took place on the 16th, and the speakers were:

Dr. William H. Welch; Dr. Stewart Paton, who was influential in securing this munificent gift from Mr. Henry Phipps; Dr. Henry D. Harlan, of the Board of Trustees; Sir William Osler, Bart, F. R. S. The rest of the program was made of scientific papers, a large part of the program being filled by distinguished scientists from Europe. The following is the program:

APRIL 16, 1913, 3 P. M.

The Henry Phipps Psychiatric Clinic.

Invocation, Rufus M. Jones, D. Litt. Minister, Society of Friends, Haverford College, Pa.; introduction, Dr. William H. Welch; "The Clinic and the Community," Dr. Stewart Paton; "A Word of Appreciation," Henry D. Harlan, President of the Board of Trustees of the Johns Hopkins Hospital; "Specialism in General Hospitals," Sir Wm. Osler, Bart., F. R. S., etc.; inspection of the building; tea.

APRIL 17, 1913, 10 A. M.

The recreation room of the Henry Phipps Psychiatric Clinic.

"The Sources and Direction of Psycho-Physical Energy," Prof. W. McDougall; "Autistic Thinking," Prof. E. Bleuler; "Personality and Psychosis," Prof. A. Hoch; "The Personal Factor in Association Reactions," Dr. L. F. Wells.

4:30 P. M.—Visit to the Sheppard and Enoch Pratt Hospital; tea.

8:30 P. M.—Meeting at Osler Hall, Medical and Chirurgical Faculty building, 1112 Cathedral street.

"A Study of the Neuropathic Inheritance in Relation to Insanity," Dr. F. W. Mott, F. R. S.; "Pellagra," Prof. O. Rossi; "Psychic Derangements Associated with Ductless Gland Disorders," Prof. H. Cushing.

APRIL 18, 1913, 10 A. M.

The recreation room of the Henry Phipps Psychiatric Clinic.

"Primitive Mechanisms of Individual Adjustment," Dr. S. Paton; "Demenz Probleme," Prof. Heilbronner; "The Interrelation of the Biogenetic Psychoses," Dr. E. Jones; "The Prognostic Significance of the Biogenetic Psychoses," Dr. G. H. Kirby.

1 P. M.—Luncheon at the Johns Hopkins Hospital.

2:30 P. M.—The recreation room of the Henry Phipps Psychiatric Clinic.

Address, Dr. Achucarro; "Anatomical Border Line Between the So-called Syphilitic and Metasyphilitic Disorders," Dr. C. B. Dunlap; "Disorders Connected with Anemia," Prof. A. M. Barrett; closing address, Prof. A. Meyer.

A remarkable feature of the entire three-days' program was the intense interest manifested by the general public of Baltimore, which speaks well for the high position which the University holds in the city and state, although it may be fairly well assumed that a part of the enthusiasm which crowded the lecture rooms to the doors, even when the programs were entirely technical, may have been due to the extraordinary respect and appreciation which Baltimore holds for Dr. Osler, who had returned to Baltimore for this occasion, from his home in Oxford, England. The citizens of Baltimore gave a large dinner at the Hotel Belvedere in honor of the occasion, at which there were present about 350 people. Dr. William H. Welch presided and, of course, the guest of honor was Mr. Henry Phipps. As to the aim and status of the Clinic, the following editorial by Dr. Henry M. Hurd, now Secretary and Adviser of the Board of Trustees, and formerly Superintendent of the Hospital, published in the *Baltimore Sun,* may be of interest:

"The opening of this Clinic in connection with the Johns Hopkins Hospital calls attention to a new method of dealing with mental disease in its early stages in Baltimore. Maryland in the past, it is true, has not been lacking in praiseworthy efforts to solve the problems which have arisen in the care of her insane. More than three-quarters of a century ago Dr. Richard Sprigg Stewart, almost single-handed, opened and organized the Maryland Hospital, now known as the Spring Grove Hospital, upon the actual site of the new Clinic. A few years later Dr. W. H. Stokes assisted the Sisters of Charity in the establishment of what is now the Mount Hope institution, and not many years afterward Moses Sheppard made the largest single bequest which had ever been made in the United States to found a similar institution, the present Sheppard and Enoch Pratt Hospital. All of these institutions for many years past have done excellent work in caring for patients suffering from the various forms of mental disease.

"The new Phipps Clinic is an attempt to apply the methods and conditions under which acute diseases are treated in a general hospital to the care and treatment of insanity. It is hoped that the effect of this will be to secure prompt admission for all patients without legal formalities or vexatious delays, just as pneumonia, typhoid fever or any other acute diseases are received for treatment at any hospital as soon as the necessity of hospital care is apparent.' The development of one of these diseases and the need of treatment are all that are requisite to secure immediate admission to a hospital. A similar ease of admission places mental disease upon the same footing as other acute diseases and a resort to a general hospital for treatment will no longer bring a stigma as to an asylum. Fortunately the liberal Maryland law providing for the voluntary admission of mental patients will render it practicable for them thus to come.

"Once admitted such patients may receive the same careful study and observation at the hands of a large and experienced staff, as other hospital inmates, to ascertain the nature and causes of their malady and the best methods of cure. They are to be treated by the most approved methods and amid surroundings which will favor a speedy restoration of health. They can have prolonged baths if continuously excited, hydrotherapy if depressed or nervously exhausted, mechanotherapy and gymnastics if debilitated and appropriate occupations and diversions when they are convalescent. Medical care, good nursing and proper food will be equally available and every effort will be made by prompt and skillful treatment to prevent the disease from becoming confirmed and to hasten the period of convalescence.

"When convalescence has been fully established and the patient is permitted to return home the watchful care of the Phipps Clinic will still be over him through the agency of a member of the staff, whose duty it will be to ascertain home conditions and supply what may be lacking in the way of advice and watchful care or treatment to prevent a return of the disorder. It is thus hoped to cure speedily those who can be helped in order to prevent the great increase of chronic insanity, which is proving such a burden to every State of the Union. This will mean much to Baltimore and Maryland, as from the nature of things the majority of patients will come from the immediate vicinity of the Phipps Clinic.

"The utilization of the Clinic for the instruction of medical students and of physicians generally in the diagnosis and treatment of insanity will be of great service to the country at large. Here are large and finely equipped laboratories for the diagnosis and study of insanity in every stage under the supervision of skilled men and an ample medical staff to engage in the work of instruction. It is confidently expected that the opening of this Clinic will mark a new development in the hospital treatment of mental diseases."

---

MEDICAL EDUCATION—ECONOMIC CONSIDERATIONS. On account of the large investment of time and expense in training men for medical practice, theoretically, every man who matriculates, should graduate—and practice. In 1911, there was about one graduate for every five students; in 1912, the ratio was almost the ideal one of 1:4, but this was due largely to diminution in the freshman classes—in itself a favorable factor.

It costs about $480 a year in the average Class A medical school for the training of each student, for which the student pays an average fee of $125.

Sectarian medicine is now practically limited to homeopathy, whose colleges have dropped from 22 in 1900 to 10 in 1912, the attendance having fallen from 1909 to 827 and the annual graduates from 120 to 185. Of eclectic schools only six are extant, which graduated 92 men in 1912, leaving only about 200 undergraduate students in the present three upper classes. Physiomedical and nondescript schools are a dead issue. It is obvious that a further shrinkage in eclectic schools must follow shortly, and the status of the homeopathic colleges is, in the main, such that the nominal objection to division of the profession amounts to little practically, even if it were important numerically.

The four-year course is universal and the five-year course is in sight. Only a very few weak schools exist whose standards of education would not have been considered ideal in 1900. With minor exceptions, the preliminary of a high school education is an established fact, and several states and a considerable number of colleges insist on at least a partial college course, while nearly one graduate in every six actually holds a college degree.

---

### Centenarians.

Mrs. Polly Trinkle died March 11, 1913, at Bristol, Tenn., aged 113.

Thomas Sullivan of Williams Bay, Wis., celebrated his 112th birthday March 10, 1913, by swimming in the bay. He claimed that this was his 100th annual swim. He is the keeper of a summer resort.

Mrs. Barbara Ann Dillinger of Stoneboro, Pa., a real daughter of the Revolution, died March 25, 1913, aged 104, after a short sickness.

Rev. P. Carylon of Falmouth, Eng., has recently celebrated his 100th birthday.

Alexander Daylight, an Indian of Kettle Falls, Wash., died about March 1, aged 114.

Milo Warrick and his wife of East Liverpool, O., celebrated their 80th wedding anniversary March 18. Mr. Warrick is 100 years old, his wife a few years younger.

Samuel Keefer, a farmer near Penn Yan, N. Y., has passed his 103d birthday. He is the oldest living graduate of the Albany Normal School. His sole attack of illness was from blood poisoning ninety years ago. He is still active and does most of his reading without glasses.

Jean Phillippe Vallee of Bar-le-duc, Belgium, a veteran of the Revolution of 1830, is living at the age of 104.

Maria Tom, a gypsy of Savannah, Ga., is in good health, in spite of having smoked practically all her life, at the age of 109.

James Ward, a native of Ireland, died in the Industrial Home of St. Catherines, Ont., April 8, aged 106, after only a week's illness.

Mrs. Martha Bowers died at the State Odd Fellows' Home of Lockport March 18, aged 102.

Rev. David J. Higgins, a retired clergyman living near Los Angeles, holding bachelor's, master's and D. D. degrees, has applied for admission to Hamline College to secure the degree of Ph. D. He is over 90.

The following notes are from the *Boston M. and S. Journal*:

John Munsinger of Howard, Kan., is said to have been born on Dec. 14, 1812, and to have already 144 living descendants. He attributes his excellent health to his moderation in all things. He has never tasted alcoholic beverage, but has smoked tobacco temperately for the past 80 years.

Charles Weidner of Sparkhill, Rockland county, N. Y., is said to have been born on March 14, 1811. He can still read without glasses. He eats sparingly, and never uses alcohol or tobacco.

Jean Boudin of Toronto, Canada, is locally reputed to be 122 years old. He has one living great-granddaughter. His longevity he ascribes to a diet of baked apples, brown bread, and boiled milk. He drinks no alcohol, but smokes tobacco freely.

Mr. W. Allison, a veterinary surgeon, who died recently at Harrowgate, England, is said to have been born on Dec. 4, 1812.

Rev. Abraham Isaac Trager, a rabbi who died recently at Charleston, S. C., is said to have been born in 1807 in Russia. He migrated to New York in 1850. He is survived by two aged daughters, 23 grandchildren and 30 great-grandchildren.

---

An American College of Surgeons was organized in Washington May 5. Four hundred and fifty prominent surgeons of North America came together at the invitation of an organization committee appointed by the Clinical Congress of Surgeons of North America in November, 1912, consisting of Edward Martin of Philadelphia, Emmet Rixford of San Francisco, John B. Murphy of Chicago, Rudolph Matas of New Orleans, Albert J. Ochsner of Chicago, Charles H. Mayo of Rochester, Minn., Frederic J. Cotton of Boston, George Emerson Brewer of New York City, J. M. T. Finney of Baltimore, W. W. Chipman of Montreal, George W. Crile of Cleveland and Franklin H. Martin of Chicago.

Each large university city on the continent was visited by a member of the committee who met a group of selected men. Of 500 invited to the meeting in Washington 450 responded, representing all branches of surgery, and were designated Founders of the College.

The call of the meeting summarizes the work for which the committee was authorized:

"1. It should formulate a minimum standard of requirements which should be possessed by any authorized graduate in medicine, who is allowed to perform independently surgical operations in general surgery or any of its specialties.

"2. It should consider the desirability of listing the names of those men who desire to practice surgery and who come under the authorized requirements.

"3. It should seek the means of legalizing under national, colonial, state or provincial laws, a distinct degree supplementing the medical degree, which shall be conferred upon physicians possessing the requirements recognized by this law as necessary to be possessed by operating surgeons.

"4. It should seek co-operation with the medical schools of the continent which have the right to confer the degree of M. D., under the present recognized standards, and urge these colleges to confer a supplementary degree on each of its graduates who have, in addition to their medical course, fulfilled the necessary apprenticeship in surgical hospitals, operative laboratories and actual operative surgery.

"5. It should authorize and popularize the use of this title by men upon whom it is conferred, and its use should especially be urged in all directories of physicians in order that the laity as well as medical men can distinguish between the men who have been authorized to practice surgery, and those who have not."

"The net result of the committee's efforts is that five hundred surgeons of all specialties, representing every large center of population, every important university city with a teaching faculty of medicine, every special and general society representing a specialty of surgery, all the important surgical clinics and hospitals, besides many independent surgeons from all portions of the North American continent, have consented to become founders of the organization under contemplation, and of this five hundred fully four hundred and fifty are here at this hour ready to fulfill their obligation."

BY-LAWS.

I. The NAME of the corporation is the College of Surgeons.

II. The OBJECT of the College shall be to elevate the standard of surgery, to provide a method of granting fellowships in the organization and to formulate a plan which will indicate to the public and the profession that the surgeon possessing such a fellowship is especially qualified to practice surgery as a specialty.

III. ORGANIZATION. The corporation is to be known as the College. The College shall consist of all members of the corporation, to be known as Fellows, and shall vest the general management of the corporation in a Board of Governors, and the Board of Governors shall in turn vest the details of the management in a board of trustees, to be known as the Board of Regents.

IV. ADMINISTRATIVE PLANS. 1. The Board of Governors shall consist of the five hundred surgeons invited by the Organization Committee to serve as founders of the College and who have signified their willingness to act in that capacity. The individuals of the first Board of Governors shall also be known as the founders of the College of Surgeons.

This original Board of Governors shall be divided into three classes, to serve one, two and three years. At the annual meeting in 1914, and at each succeeding annual meeting, the Fellows of the College shall elect fifty surgeons to membership in the Board of Governors, each for a term of three years. Thirty of these are to be elected from a list of nominations consisting of two members each, nominated by the following surgical societies and associations of North America:

American Surgical Association. Section on Surgery of the American Medical Association. Section on Obstetrics, Gynecology and Abdominal surgery of the American Medical Association. General Surgical Division of the Clinical Congress of Surgeons of North America. Division of Surgical Specialties of the Clinical Congress of Surgeons of North America. American Gynecological Society. Southern Surgical and Gynecological Association. Western Surgical Association. Section on Surgery of the Canadian Medical Association. American Association of Obstetricians and Gynecologists. American Orthopedic Association. American Association of Genito Urinary Surgeons. American Laryngological Society. American Ophthalmological Society. American Otological Society.

Twenty members shall be elected at large to represent surgeons of North America not affiliated with the above societies or associations.

The Board of Regents shall consist of twelve surgeons, members of the Board of Governors, elected by the Governors, these to be divided into three classes whose terms of service shall expire in one, two and three years. Their successors shall be elected each for a term of three years. Not more than three of each class of four shall be elected from one country. The Board of Regents is increased to fifteen in number by three officers of the Corporation, the President, Treasurer and General Secretary. The two Vice-Presidents are ex-officio members of the Board. The Board of Regents is the administrative body of the corporation, corresponding to a board of trustees in other corporations.

V. Fellowships. The Fellows of the College shall be graduates in medicine, who are legalized to practice medicine in their states and provinces, who have made an application for fellowship, such application to be endorsed by three Fellows of the College, one of whom shall be a member of the Board of Governors, and who meet the qualification requirements that shall from time to time, be established by the Board of Regents, and who shall be elected to fellowship by the Board of Regents on recommendation of the Committee on Credentials.

All Fellows of the College shall be designated a Fellow of the College of Surgeons and shall be authorized and encouraged to use the letters F. C. S. after his name on professional cards, in professional directories and in scientific articles published in surgical literature.

VI. Fees. An initiation fee of Twenty-five Dollars shall be required of each member of the College on his election to fellowship by the Board of Regents. The annual dues will be Five Dollars.

VII. DIRECTORY. The Board of Regents shall issue each year a directory containing the names and addresses of the Fellows of the College of Surgeons, arranged by states, provinces and colonies.

VIII. EXPULSION. Any member of the College may be expelled for unprofessional or other conduct inconsistent with the rules and regulations of this Corporation by a majority vote of the Board of Regents.

IX. STANDING COMMITTEES. The Board of Regents shall elect the following standing committees: 1. Credentials. 2. Legislation. 3. Graduate Schools and Hospitals.

These by-laws were unanimously adopted with the provision that the Board of Regents should make any minor corrections deemed desirable and present such corrections for adoption at the next meeting of the Board of Governors.

### OFFICERS ELECTED.

President, J. M. T. Finney, Maryland; First Vice-President, W. W. Chipman, Quebec; Second Vice-President, Rudolph Matas, Louisiana; Treasurer, A. J. Ochsner, Illinois; General Secretary, Franklin H. Martin, Illinois.

### BOARD OF REGENTS.

J. M. T. Finney, Maryland; A. J. Ochsner, Illinois; Franklin H. Martin, Illinois; George E. Brewer, New York; George E. Armstrong, Quebec; John B. Murphy, Illinois; Edward Martin, Pennsylvania; F. J. Cotton, Massachusetts; Herbert A. Bruce, Ontario; C. F. Stokes, Washington, D. C.; William D. Haggard, Tennessee; George W. Crile, Ohio; Robert E. McKechnie, British Columbia; Charles H. Mayo, Minnesota; Harry M. Sherman, California.

### SELECTION OF FELLOWS.

The prospective Fellows are to be divided into four classes. Classes A, B and C are to be admitted without the formality of submitting to an examination.

The A class shall consist of founders of the College.

The B class shall consist of the members of the special surgical societies constituting the Congress of American Physicians and Surgeons and one hundred each, nominated by accredited committees, from the Surgical Section of the American Medical Association, from the section on Obstetrics, Gynecology and Abdominal Surgery of the American Medical Association, from the General Surgical Section of the Clinical Congress of Surgeons of North America, from the Division of Surgical Specialties of the Clinical Congress of Surgeons of North America, from the American Association of Obstetricians and Gynecologists, from the Surgical Section of the Canadian Medical Association, from the Southern Surgical and Gynecological Association and from the Western Surgical Association.

The C class shall consist of surgeons of prominence of five years in the practice of surgery or a surgical specialty and who, in the opinion of the Committee on Credentials, are eligible for fellowship in the College without formal examination.

For all others, coming under Class D, it was resolved that the Board of Regents, through the Committee on Credentials, limit the admission of the Fellows to classes A, B and C until the Board of Regents formulates a standard of requirements for class D and reports the recommendations back to the Board of Governors for approval at the meetings to be called by the Board of Regents in Chicago, November, 1913.

APPLICATIONS FOR FELLOWSHIPS.

It will be the spirit of this Association to open the fellowship to all competitors in surgery without favor. Scientific attainments, surgical ability, unquestioned moral character, measured by the College's standards, shall constitute the measure for fellowship.

There are many hundreds of surgeons on the continent who are not included in classes A and B, who fall into the C class. Applications from these men will be welcome and their names will have the most careful consideration by the Committee on Credentials.

All applications for membership should be forwarded to the Secretary of the corporation. It would add to the ease of the work of the Committee on Credentials if references in the way of vouchers or recommendations from one or more well known surgeons accompany each application for fellowship.

FORMAL CONFERRING OF FELLOWSHIPS.

The first convocation for the formal conferring of fellowships will occur in November, 1913, at a time and place that will be designated later. The first directory of Fellows will be distributed at that meeting. For that reason the applications for fellowships on the part of A, B and C classes should be filed as promptly as possible in order to facilitate the correcting of lists for publication.

We publish the foregoing announcement almost in full, as it deserves the careful attention of the profession. Specialism has never, hitherto, been officially and legally recognized, but has depended upon individual effort and the achievement of personal recognition of skill. If this plan succeeds, it will afford a precedent for the future establishment of standards of admission to other specialties. The initiators of the American College of Surgeons seem to have foreseen and to have avoided the establishment of a professional aristocracy, so far as is consistent with the formulation of necessary standards of admission. Specialism is inevitable with the perfection and extension of any branch of science and art. It is obviously liable to be exploited for private ends, if not properly controled, and, on the other hand, there is already an unfortunate tendency on the part of certain specialists to regard themselves as more or less apart from the general profession of medicine. This tendency must be avoided in the interests of professional solidarity. A very delicate problem in the formal recognition of specialization is to provide for the transition stage from general to special practice—and we believe that every specialist in a practical branch should have experience of a few years in general practice. Another equally difficult problem is that of the man who, by reason of such circumstances as small population or personal choice, wishes to deal with a certain specialty without abandoning general practice. This problem is especially difficult because it is impossible to distinguish formally and legally between the sincere and well qualified general practitioner with a hobby and the mercenary dollar-hunter equipped with a double-barreled gun for all classes of game. Still another problem is to provide for necessary attendance on emergent surgical cases by general practitioners when the delay of obtaining an accredited surgeon would outweigh the greater risk of operation by one relatively inexpert, and for distinction between cases requiring special skill and those of minor nature properly included in general practice.

In no hostile spirit it may be suggested that the College of Surgeons should clearly recognize that it is a poor rule that does not work both ways. It is of common occurrence that surgeons, perfectly competent as operators, overlook points in alimentary and other branches of physiologic chemistry, in methods of diagnosis, in ordinary physiology, etc., so that their work results quite as fatally as an operation undertaken by a physician without proper skill and equipment. Any formal limitation of special work to a special branch of the profession must recognize that specialism is exclusive as well as inclusive.

---

PROPOSED A. M. A. SECTION ON GASTRO-ENTEROLOGY AND PROCTOLOGY. Dr. Anthony Bassler, 126 E. 60, N. Y., is circulating a petition for the establishment of such a section. There is much to be said for and against this proposition. From one standpoint it is undesirable to duplicate the special functions of the respective associations already in existence; from another, the exclusiveness and small numbers of these associations leaves a need for such a section open to all members of the A. M. A. Embarrassment might be caused if the members of these organizations took an active part in the more popular section while maintaining their associations of limited membership, while bad feeling might result if the latter held aloof from the new section if it be formed. From quite a different viewpoint the question must be considered just how far it is profitable and desirable to break up the great national gathering into sections and whether except in the case of well established and numerically strong specialties it is worth while to pass beyond the line of considering special problems in the general medical and surgical sections and to substitute for the joint meetings of certain sections a definite section confining itself to the medical, surgical and chemic problems of this or any other phase of medicine. These questions can be best answered by the actual results obtained by circulating the petition, provided that those favoring it realize fully that they are committing themselves to a separation of interests from those of the more general sections already existing.

---

A Typhoid Epidemic has followed the inundation of Albany's filtration plant during the flood late in April.

---

Seizure of Cocaine. Eight hundred pounds, valued at $50,000, smuggled from Mexico, was seized in San Francisco, April 8.

---

Amalgamation of Medical Schools. The expected merger of the Baltimore Medical College and the Medical Department of the University of Maryland has been officially announced.

---

Civil Service Examinations. We received during the first week of May announcements of examinations for positions as physicians, dentist and dental interne, to be held June 4. It is impossible for a monthly journal appearing about the first of the month to make such announcements in time for candidates to secure the necessary papers, and we urge the Civil Service to give longer notice of future examinations.

---

Army Medical Corps Examinations will be held at places to be designated later, on July 14. Applications should be made to the Surgeon General, U. S. Army, Washington, D. C. There are at present forty vacancies to be filled. Successful candidates will be commissioned First Lieutenant in the Army Medical Corps.

Automobiles killed 967 and injured 6107 persons in New York State, last year.

---

Cancer Serum. That recently announced by Dr. Howard W. Nowell of Boston University has produced immunity and apparently cures of implanted human cancer in rabbits. Fifty patients have shown favorable immediate results. It is to Dr. Nowell's credit that he disclaims a positive cure and that he expressly advises against reliance on the serum in operable cases.

---

Historical Medical Museum. The Historical Medical Museum, organized by Mr. Henry S. Wellcome, which is to be opened in London towards the end of June next, will include some objects of exceptional historical medical interest.

An important exhibit in the science section will be a large collection of the original apparatus used by the famous Galvani in making his first experiments in Galvanism in the eighteenth century.

A remarkable collection of votive offerings for health will be exhibited. The custom of presenting these offerings in cases of sickness is a very ancient one, and the collection that will be shown is probably the finest ever brought together. It will include Graeco-Roman votive offerings of special anatomical and pathological interests in silver, bronze, marble and terra cotta, together with a number of similar objects used for the same purpose in medieval and modern times.

Ancient microscopes and optical instruments, gathered from all quarters of Europe, will form another important feature, and a selection of surgical instruments used by famous surgeons when operating on historical personages is promised.

The collection of amulets and charms connected with English folk medicine will be very complete, and will constitute an exhibit of more than ordinary interest.

A fine collection of early medical medals and coins from the Graeco-Roman period, ancient manuscripts and early printed medical books, will also be shown, together with many other objects of interest to medical and scientific men.

---

Consumption of Sugar. Excepting that the extensive industry of preserving for export makes the nominal per capita consumption of sugar about three pounds more for Great Britain, the United States leads the world in the use of sugar—nearly forty pounds. This equals an average daily consumption of a trifle over fifty grams, which is well within physiologic limits.

Friedmann Vaccine. It is scarcely necessary to comment on the marked contrast between Nowell's and Friedmann's announcements and methods. It is worth while, however, to emphasize the fact that the ethical issues connected with the latter are by no means so important as the actual results obtained. In view of the recent unfavorable report of the Public Health Service, we beg leave to reiterate our statement made a couple of months ago, that at least two years must elapse before a final decision can be reached. A discovery may fall far short of being a positive cure and yet have enormous value, especially after accumulated experience enables a better judgment of appropriate cases, of dosage, etc.

---

Legal Status of Talking in Sleep. The Supreme Court of Colorado has recently decided that a confession by this means does not hold, on the ground that it is not voluntary. The prisoner, charged with murder, repeated in his sleep the words, "I killed her." It should also be considered that aside from the legal technicality involved, such a confession really means nothing. The theory has recently been advanced that ambidexterous persons do not dream. The writer is nearly ambidexterous and nearly dreamless, but, in a limited personal experience and a considerable second-hand experience drawn from many sources, dreams by no means represent actual occurrences. The words of the prisoner undoubtedly indicated that, in his sleep, he dreamed of being a murderer. Under the strong suggestion of criminal proceedings, an innocent man would be just about as likely as a guilty one to use the language mentioned.

---

Prescriptions in English. A bill has been introduced into the Nebraska legislature by Mr. Smith, which provides that English shall be used in the writing of all prescriptions, and forbids the use of any other language. A similar bill has been introduced into the North Dakota legislature by Mr. Davidson.

While it is the obvious intention of this bill to prevent mistakes, it must be borne in mind that the terms coined for many synthetics are scarcely English, and no more simple without the genitive ending than with it. Moreover, the use of English words for the older *materia medica* would, in many instances, lead to confusion, whereas the Latin term is definite and official.

---

Analyses of Niagara River Water are being carried on by the national and state health authorities, with the co-operation of various local health boards, in order to solve some of the problems of water supply and sewerage of the Niagara Frontier.

Osteopaths Allowed to Sign Death Certificates. This action was taken by the N. Y. City Board of Health, May 7. This strikes us as logical. If a special kind of practitioner is allowed to treat disease, let him assume the responsibility of death. Moreover, the older we grow, the more we favor the personal liberty of every adult, legally compos mentis, to make his own decisions, even if wiser persons regard them as detrimental to his own welfare. And, the more we favor competition, not by exclusion, but by making good along our own lines. Of course, this does not imply that we believe in allowing everyone to jeopardize the health of the community.

---

The Buffalo Educational Union now includes the University of Buffalo along with such other educational institutions as the Public Library, Art Gallery, Historical Society, Society of Natural Sciences, and Grosvenor Library. This is in line with the Chicago idea, favorably noted in our columns last year. Perhaps the systematic co-operation of the various educational institutions now available may yet solve the problem of a collegiate department. We have the libraries, museums, an available teaching force, exceptional advantages for field work along certain scientific lines and for many branches of engineering. All that is needed is enough tainted money to crystalize these into a college. And we bespeak similar educational unions for other cities in our territory and even the smaller towns.

---

Dartmouth College Medical School, established in 1798, and fourth in age in the country, will be discontinued except for preparatory courses, after the graduation of the class of 1914. This voluntary dissolution, due to the impossibility of maintaining modern standards without the clinical material of a large city, is a commendable precedent.

---

Pigs With Uncloven Hoof. A. L. Benedict of New Canaan, Conn. (probably a distant cousin of the Editor), and A. V. Barnes, of the firm of A. S. Barnes & Co., each have litters of pigs showing this peculiarity in about half the number. These litters were bred from a similar freak sire discovered in Ohio by the late Dr. P. H. Hiss, the bacteriologist. This exemplification of the Mendelian law is interesting. It is said that such pigs are immune to hog cholera.

---

The graduating exercises of the Training School of the Buffalo Homeopathic Hospital will be held June 3 at the Assembly Room of the Hospital.

The Travel Study Tour of American Physicians to the XVII International Congress of Medicine will sail from New York on July 3d, on the North German Lloyd steamship "Bremen." About 75 physicians will participate in this tour, the chairman of which is Dr. W. B. De Garmo, New York City, Secretary, Dr. Richard Kovacs, 236 East 69th street, New York. In co-operation with the International Committee for Postgraduate Medical Education, arrangements have been made to visit Dresden, Berlin, Cologne, Brussels, etc. (clinics and hospitals at Paris, Munich, Vienna), and inspect the health resorts of Carlsbad, Marienbad, Nauheim, Hamburg, Wiesbaden. No American party ever enjoyed similar privileges. The party will finally attend the International Congress of Medicine August 6th to 12th in London.

---

New Powers to State Health Department. Gov. Sulzer has signed the bill dividing New York State into twenty health districts, each with a medical officer, and providing for the inspection of cold storage plants, hotel and restaurant kitchens, and otherwise increasing the powers of the State Health authorities.

---

Prevention of Dust in Buffalo. Health Commissioner Fronczak has formulated an ordinance which, if passed, will forbid the beating of rugs and emptying of dust out doors. Male posterity will doubtless erect a monument to him as the man who robbed house cleaning of its terrors. The ingenuity of housewives, who cannot afford to send carpets and rugs to steam cleaning works, will be taxed to devise means not to let the dirt accumulate indoors.

---

Railroad Rates to Medical Conventions. The rather illiberal treatment of the medical profession in this respect leads us to inquire why physicians should not have the same rates as Sunday School teachers, fraternal organizations, commercial associations and others. But, better than a demand for special concessions, would be the universal inforcement of a 2-cent rate all over the country, with a cent and a-half rate for any round-trip completed within two weeks, either in the ordinary sense of a passage back and forth between two points, or in the more correct sense of a loop-trip (rundreise), with the privilege of choice of routes and stop-overs, with and without formality, as in Europe.

